# 512. Contact Burn Wound Zones of Necrosis Are Histopathologically Different from Converted Zones of Stasis

**DOI:** 10.1093/jbcr/irag033.171

**Published:** 2026-04-06

**Authors:** Abigail Bruhm, Kai V Myree Lindsey, Lian Daniel N Manalo, Amanda Soo Ping Chow, Alina A Watson, Bonnie C Carney, Lauren T Moffatt, Jeffrey W Shupp

**Affiliations:** The Burn Center at MedStar Washington Hospital Center, Washington, DC; The Burn Center at MedStar Washington Hospital Center, Washington, DC; The Burn Center at MedStar Washington Hospital Center, Washington, DC; The Burn Center at MedStar Washington Hospital Center, Washington, DC; Georgetown University, Washington, DC; The Burn Center at MedStar Washington Hospital Center, Washington, DC; The Burn Center at MedStar Washington Hospital Center, Washington, DC; MedStar Washington Hospital Center, Washington, DC

## Abstract

**Introduction:**

After burn injury, the conversion of the zone of stasis to necrotic tissue is described to occur within 24-72 hours. Increased inflammation, decreased perfusion, and/or apoptotic/necrotic cellular signaling have been implicated, but the extent of damage in converted tissue is poorly characterized. Burn conversion is thought to increase total body surface area and/or depth of injury increasing morbidity and mortality. Characterizing converting tissue may give insights into tailored therapies to prevent this process and optimize surgical intervention. We hypothesized zones of stasis assessed as “converted” would have less severe tissue damage by histopathology compared to the original contact area (zone of necrosis).

**Methods:**

Male Sprague–Dawley rats received a comb contact burn on each flank resulting in 4 burns and 3 unburned interspaces. Data were collected pre- and post-injury and every 24 hours for 3 days. Digital images were used to determine viable tissue areas by tracing in Image J Software, and Laser Dopler Imaging (LDI) assessed perfusion. Biopsies were processed, sectioned, H&E stained, imaged and then graded by an investigator blinded to the tissue type using a scale to determine epidermal and dermal appendage damage severity. Severity scores (1-4) were evaluated using a 2-way ANOVA. Average interspace area and perfusion were evaluated by a Wilcoxon test.

**Results:**

Viable tissue area based on digital imaging decreased significantly over 24-hours, demonstrating conversion of interspace to necrotic (76.5 ± 12.6 vs. 20.0 ± 7.8 cm2 *p*=.0078) (Fig A). Perfusion decreased significantly over the same 24-hours (245.5 ± 39.51 vs. 117.6 ± 42.47 perfusion units, *p*=.0078) (Fig B). Dermal appendage damage severity in interspace tissue was significantly lower than burn wound on days 0 and day 1 (Day 0 = (1, (1,1) vs. (3, (2,3), Day 1 = (1, (0,1) vs (3, (2,3) *p* = < 0.0001) (Fig C). The same was found in epidermal damage severity in the interspace and burn wound (Day 0 = (1, (1,2) vs. (3, (3,3), Day 1 = (1, (1,2) vs. (3, (3,3) *p* = < 0.0001) (Fig D).

**Conclusions:**

While after conversion zones of stasis appear more like the zone of necrosis compared to uninjured skin after 24 hours, they demonstrate less dermal appendage injury. Evaluation of zones of stasis could be informed by histological evaluation to assess tissue damage severity and ability to regenerate, obviating the need for excision and autologous grafting.

**Applicability of Research to Practice:**

This study may help further the understanding of burn conversion, and to what extent and over what time course the zone of stasis converts to a wound that cannot regenerate. Accurately assessing burn wound conversion in areas that were previously unburned may have implications for wound excisional area and depth, ultimately impacting the amount of autologous skin needed for definitive coverage.

**Funding for the study:**

N/A.

